# Mechanical properties of epoxy composites filled with multi-walled carbon nanotube, Nanoaluminum particles and glass fibers at elevated temperatures

**DOI:** 10.1016/j.heliyon.2024.e38232

**Published:** 2024-09-26

**Authors:** Mohammed Nadeem M, Jaimon Dennis Quadros, Yakub Iqbal Mogul, Ma Mohin, Abdul Aabid, Muneer Baig, Omar Shabbir Ahmed

**Affiliations:** a Senior Lecturer of Mechanical Engineering, University of Bolton, RAK Academic Center, 16038, Ras Al Khaimah, United Arab Emirates; b National Centre for Motorsport Engineering, University of Bolton, Deane Road, Bolton, BL3 5AB, UK; c School of Engineering, University of Bolton, Deane Road, Bolton, BL3 5AB, UK; d Department of Engineering Management, College of Engineering, Prince Sultan University, PO BOX 66833, Riyadh, 11586, Saudi Arabia

**Keywords:** Carbon nanotubes, Glass fibers, Impact strength, Toughness, Tensile strength, Temperature

## Abstract

The present work investigates the mechanical properties of a composite material composed of multi-walled carbon nanotubes (MWCNTs), nano-aluminum powder (NAP), and glass fibers (GF) for five different compositions. The study further investigated how MWCNTs contribute to maintaining the mechanical properties of nanocomposites when exposed to elevated temperatures, up to 180 °C. The evaluation of impact strength revealed that the nanocomposite, composed of 2 % MWCNTs, 15 % NAP, and 10 % GF, demonstrated the greatest impact strength. At room temperature, the composite containing 2 % MWCNTs, 5 % NAP, and 20 % GF exhibited the highest ultimate tensile strength (UTS). Conversely, at elevated temperatures reaching up to 180 °C, the highest UTS was observed in the composition with 2 % MWCNTs, 10 % NAP, and 15 % GF. The hardness of the nanocomposite was influenced by its composition; at room temperature, the maximum hardness was observed in the mixture containing 2 % MWCNTs, 20 % NAP, and 5 % GF. In contrast, at elevated temperatures, the composition with 2 % MWCNTs, 5 % NAP, and 20 % GF exhibited the highest hardness. Overall, the study found that incorporating GF and NAP improved the mechanical properties of the composite. These results indicate that the composite's performance could be further optimized for specific applications through the addition of filler materials.

## 1 Introduction

The utilization of fiber-reinforced composites has brought significant advancements in the creation of lightweight structures. Particularly, the aviation industry has recognized the substantial benefits of meeting stringent performance [1] and reliability standards. Advanced composites are increasingly being adopted in various aviation components, including fighter aircraft airframes, helicopter parts, commercial aircraft fins, satellite panels, antennas, rocket engines, and small aircraft airframes [2]. The advance of strong and rigid fibers, such as glass (S-glass) and carbon, along with advancements in polymer science [3], has played a crucial role in composite development, with carbon fibers proving particularly versatile. The complexity of newly developed polymer-based composites has been addressed through advancements in technology and computational analysis methods [4,5]. These developments have aided in analyzing and understanding the behavior of composite materials, providing predictive tools for design purposes [5]. Continuous fiber-reinforced polymer-matrix composites are widely used for structural applications requiring load-bearing capacity, with epoxy-based resins reinforced with carbon and glass fibers [6] being the most prevalent materials.

Successful applications in current aircraft designs have encouraged engineers to explore polymer composites for various industries encountering higher temperatures. This includes components near aircraft engines in present-day use and future high-speed transport aircraft. As aircraft designs incorporate higher speeds and space re-entry features, future structures, particularly leading edges, may need to endure even higher temperatures (350 °C or above). While the reinforcing fibers like glass or carbon [5] are less affected by high temperatures, the polymer matrix material requires attention. Therefore, the current research has focused on developing high-temperature polymer-matrix composites. In recent decades, research efforts have yielded polymeric matrices that perform satisfactorily up to 300 °C [7], and there are promising trends in developing polymer matrices with service temperatures up to 400–500 °C [7,8]. However, each new polymer system presents challenges in producing high-quality composite components, remaining a crucial aspect of research in high-temperature polymer-matrix composites.A wide number of studies are available wherein authors have tried to develop hybrid composite materials, using nanotubes, fibers, and filler materials. Majority of such composites see the use of CNT as they possess excellent mechanical, tribological, electrical, chemical, and thermal properties [4], which can suit a variety of applications that include structural, aerospace automotive, construction, energy storage, coatings, etc. [6,8]. Works by Kim et al. [1] investigated various alternatives for filler materials and binders, such as phenolic resin and epoxy resins. They examined the impact of these materials on the performance of brake pads. The study involved formulating different compositions of fillers, fibers, and binders to explore the possibility of replacing existing formulations and their effects on the physical and tribological properties of the brake pads. Huang et al. [2] recognized electrospinning as an efficient technique for producing polymer nanofibers, mostly in solvent solutions and some in melt form. These nanofibers hold potential applications, particularly as reinforcements in nanocomposite development. Robert and Benmokrane [3] focused on the use of fiber-reinforced polymers (FRP), specifically glass fiber-reinforced polymer (GFRP) reinforcing bars, as internal reinforcement for concrete structures. They examined the behavior of GFRP bars subjected to extreme temperatures, which is critical for applications in North America, particularly in Canada. The study investigated the thermal stability, strength, ultimate elongation, and modulus of GFRP bars. Feng et al. [4] studied carbon nanofibers (CNF), which were extensively researched due to their significance in fundamental scientific research and practical applications. CNF composites have the potential to be used in various fields, including electrical devices, battery and supercapacitor electrode materials, and sensors. Li et al. [5] discussed carbon nanotubes (CNTs), which are nanostructured allotropes of carbon. They highlighted the unique properties of CNTs that make them potentially useful in numerous nanotechnology applications. Their exceptional surface area, stiffness, strength, and resilience generated great excitement in the field of pharmacy. Chisholm et al. [9] conducted a systematic study to investigate the properties of a matrix by incorporating micro and nano-sized silicon carbide (SiC) fillers into an epoxy matrix. The study revealed that the addition of nanoparticle fillers resulted in superior thermal and mechanical properties as compared to micro-fillers. The resulting structural composites were then evaluated for their mechanical properties under flexural and tensile loads. Yu et al. [10] determined the tensile strengths of individual MWCNTs with a “nanostressing stage” located within a scanning electron microscope (SEM). The results revealed that MWCNTs broke in the outermost layer, and the tensile strength ranged from 11 to 63 GPa for the set of MWCNTs loaded. El-baky et al. [11] reviewed the compressive and flexural strength of cement mortar with varying amounts of nano-silica. They found that the strength increased proportionally with the increasing amount of nano-silica, especially at early ages. However, the strength decreased after reaching an optimum percentage of 7 %, which was attributed to the depletion of calcium hydroxide during the activation process. Ku et al. [12] investigated the use of glass powder as a filler in epoxy resin composites for structural applications. They found that composites with 25 % glass powder by weight exhibited the highest flexural strength and Young's modulus, along with reasonable fluidity during the casting process. The highest flexural strain was achieved with a glass powder content of 10 % by weight. Wetzel et al. [13] explored the addition of alumina nanoparticles to epoxy resin to improve stiffness, impact energy, and failure strain at low filler contents (1–2 vol%). They highlighted the importance of proper surface treatment to enhance the interaction between the fibers and the matrix. Furthermore, they found that adding CaSiO_3_ nanoparticles further enhanced the mechanical properties and wear resistance of the epoxy matrix. Manjunath et al. [14] improved the flexural and tensile properties of nanoclay embedded GFRP with the addition of nanoclay particles thereby, delaying their strength reduction. Ayatollahi et al. [15] studied the influence of MWCNT and nanosilica on the tensile behavior of CFRP composites. The study revealed that the addition on nanosilica improved the tensile behavior for composites with 0.5 wt% of MWCNT and nanosilica.

Apart from fiber reinforced composites, polyproplylene (PP) and polyethylene (PE) are widely used polymers in nanocomposites due to their excellent mechanical properties. Several nanoparticles and nanotubes have been impemented to these composites to enhance their mechanical performance for different applications. Dimitrios et al. [16] determined the tensile strength and tensile modulus of a CNT filled polypropylene nanocomposite. They found tat the tensile strength and modulus of CNT/PP nanocompiste increased up to a particular CNT content, however, decreased with further increase due to agglomeration of the CNTs. Xiao et al. [17] studied the tensile and impact strengths of MWCNT/PP at low speeds. The study observed that both the values increased when the MWCNT content was about 0.6 %. Zou et al. [18] investigated the impact strength of high density polyethylene (HDPE)/MWCNT nanocomposites, and found that, with the addition of MWCNT, the impact strength increased suggesting a good load transfer effect. According to Xiao et al. [19], when the wt% of MWCNT was maintained above 10 %, the tensile strength increased by 56 % for MWCNT/low desity polyethylene (LDPE) nanocomposite.

Analyzing nanotube-composite interfacial dynamics efficiently is the major emphasis of our investigation since it has a large impact on load transfer efficiency and mechanical integrity of the nanocomposite. It is necessary to have a better knowledge of their interactions to benefit the broader growth of nanotechnology and nanomaterial development. Bal et al. [20] emphasized the importance of effectively dispersing CNTs in polymers to ensure homogeneity and maximize filler surface area. They proposed chemical functionalization of CNTs to enhance their interaction with the polymer matrix. Covalent bonding between the polymer and CNTs improved interfacial interactions, resulting in better mechanical performance. The authors stated that covalent bonding had a negligible influence on the mechanical properties of CNTs. Hammel et al. [21] discussed the utilization of CNFs in composite materials. They acknowledged that CNFs might have imperfect structures compared to other CNTs but are more feasible for industrial production due to the required volume. The authors highlighted the significance of appropriate surface treatment of CNFs to enhance their interaction with the matrix. They found that adding CaSiO_3_ nanoparticles further improved the mechanical properties and wear resistance of the epoxy matrix. CNTs already possess excellent mechanical properties that ensure efficient load transfer [5]. Its hybridization with fibers and nanoparticles develops a network that uniformly distributes load across the composite [13]. This improves the overall strength of the composite and its ability to resist deformation. According to Shireesha and Chidurala [22], nanoparticles such as graphene, etc., contribute to thermal and electrical conductivity. As they interact with the CNTs, the composites’ overall heat dissipation and thermal expansion is largely reduced.

According to the literature, several researchers worked on the manufacturing and development of hybrid composite materials, including CNTs as well as other fibers and fillers, hence improving their overall productivity. However, insufficient research has seen the usage of aluminum nanoparticles as fillers in composite materials. Previous research shows that aluminum nanoparticles act as catalysts in various chemical reactions due to their high surface area and reactivity [23]. They have also been incorporated into materials, such as, plastics and metals, to improve their mechanical, thermal, and electrical properties [[23], [24], [25]]. Additionally, aluminum nanoparticles are used in coatings to provide improved corrosion resistance, UV protection, and antimicrobial properties [24]. These distinct features have encouraged the incorporation of aluminum nanoparticles in composites to further investigate their applications. Additionally, the combination of MWCNT, aluminum nanoparticles, and GFs for any type of composite is hardly investigated. The current study, therefore, intends to determine the mechanical properties, such as impact strength, fracture toughness, ultimate tensile strength (UTS), and hardness of the composite material composed of five different compositions of multi-walled carbon nanotubes (MWCNTs), nano aluminium powder (NAP), and glass fibers (GF).

## 2 Fabrication and testing of composites

Five distinct composite compositions were developed by adjusting the proportions of MWCNTs, NAP, and GFs to prepare the nanocomposite. The specific content of each composite composition in terms of percentage (%) and weight, and their physical and mechanical properties could be found in Table 1, Table 2, respectively.

**Table 1:** Composition of specimens.

Composition by weight	Percentage (%)				Grams (gms)			
Specimen No.	1	2	3	4	1	2	3	4
Epoxy Resin – LY556	66.36	66.36	66.36	66.36	94.8	94.8	94.8	94.8
Hardener – HY951	6.63	6.63	6.63	6.63	7.80	7.80	7.80	7.80
MWCNT	2	2	2	2	0.74	0.74	0.74	0.74
NAP	5	10	15	20	24.0	48	72.1	96.1
Glass Fibres	20	15	10	5	60.4	45.3	30.1	15.1
Total	100	100	100	100	187.7	196.6	205.5	214.5

**Table 2:** Physical and mechanical properties of the nanocomposite constituents.

Material	Property	Value
MWCNT	Production method	Chemical vapor deposition
	Diameter	13–16 nm
	Surface area	275–330 m^3^/g
	Length	Avg. 20 μm
	Nanotubes purity	>98 %
	Tensile strength	35–180 GPa
	Thermal conductivity	6000 W/m-K
	Electrical conductivity	6000 S/cm
	Youngs Modulus	1.5–2 TPa
Glass fibers (GF)	Density	2550–2700 g/m^3^
	Tensile strength	1.95–2.05 GPa
	Thermal conductivity	1.2–1.35 W/m-K
	Electrical conductivity	6000 S/cm
	Poisson's Ratio	0.21-0.23
	Youngs Modulus	2.75–2.9 GPa
Nano-aluminum powder (NAP)	Density	2700 g/m^3^
	Particle size	10–20 nm
	Purity (Alumina content)	>98 %
	Thermal conductivity	247 W/m-^0^C
	Electrical conductivity	6000 S/cm
	Youngs Modulus	70 GPa

### 2.1 Preparation of the composites

**Fig. 1:**
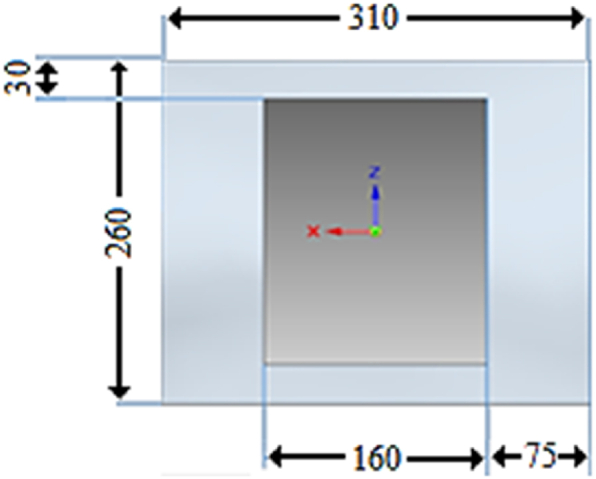
Design specification of the mould plate (mm), thickness t = 3 mm.

The initial step involves weighing the necessary materials, which are then combined and thoroughly mixed. The resulting mixture is then poured into a prepared mould with the desired thickness as per the requirement, as depicted in Fig. 1. It is vital to note that resin and hardener start to solidify approximately 30 min after mixing, indicating that the composition should be poured into the mould before the specified setting time. The plates along with mould are then compressed using a hydraulic press. Additionally, other prepared compositions are poured into moulds by adjusting the proportions of NAP and GFs while keeping the other weights (%) constant. The NAP and GF contents are varied in increments of 1 %, ensuring that the combined sum of NAP and GFs remains at 25 % of the total weight in each composition. The moulds are allowed to solidify and cure at room temperature for about 12–14 h. After solidification, the specimens are removed from the moulds and subjected to post-curing in a hot air oven at 100 °C for 12 h. Post curing is conducted at a temperature slightly below the glass transition temperature (T_g_), to relieve internal stress and improve strength and hardness of the nanocomposite. The specimens are subsequently extracted and labelled according to the respective test standards. Specific specimens are prepared for conducting tests on tensile strength, impact strength, and hardness, respectively. The texture and, morphology and worn-out surfaces of the samples were evaluated using a scanning electron microscope (SEM) (JSM-6380LA, JEOL, Japan).

### 2.2 Tensile testing

Tensile tests were conducted in accordance with the ASTM D-638 standard [26]. A computerized Universal Testing Machine (UTM) was utilized for this purpose. The dimensions of the tensile specimen were 165.0 mm × 19.0 mm x 3.2 mm, with a gauge length of 50 mm. The results obtained from these tests were used to calculate the tensile strength of the composite samples. The tensile strength of the samples exhibited a mean value with a standard deviation of ±0.80 MPa, indicating low variability across the tested specimens.

### 2.3 Impact testing

Composite specimens with V-notches were subjected to Izod impact tests according to the ASTM D256 standard [26]. To conduct these tests, an impact tester was employed. The specimens had dimensions of 64.0 mm × 12.0 mm x 3.2 mm. The impact energy values for different specimens were directly recorded from the digital indicator and reported. The impact energy of the samples exhibited a a standard deviation of ±0.0015 J, indicating low variability across the tested specimens. Table 3 shown below summarizes the key specifications related to the pendulum impact tester.

**Table 3:** Impact testing machine specification.

SI. No.	Parameters	Units
1	Potential energy	300 J
2	Pendulum drop angle	140 (degrees)
3	Pendulum strking velocity	5.175 m/s
4	Pendulum weight	22.45 kgs
5	Angle of striking edge	30 ± 20 (degrees)
6	Radius of striking edge	8 ± 0.05 (mm)

### 2.4 Hardness testing

The hardness of the specimens was evaluated using the Shore-D method, following the ASTM D2240 standard [10,26]. A Durometer was employed for these tests. The Durometer was placed on the specimen, and pressure was applied to ensure proper contact between the tester's flats and the specimen surface. Readings were directly obtained from the dial, and subsequently, the specimens were subjected to different temperatures, and readings were recorded to analyze the changes in hardness with temperature. The hardness readings exhibited a standard deviation of ±3 on the scale indicating low variability.

### 2.5 Wear testing

The wear test is conducted using a pin-on-disc machine. In this setup, sliding occurs between a stationary pin or ball and a rotating disc, at a predetermined speed. The disc is powered by a robust 2068 HP motor capable of running at speeds ranging from 0 to 3000 rpm. Exact loading could be achieved by either manual operation or software automation. A piezoelectric sensor is employed to measure the frictional force. The specifications of the pin on disc apparatus are shown in Table 4. Composite specimens were subjected to wear tests according to the ASTM G-99-05 standards [27,28]. The specimens had dimensions of 10.0 mm × 10.0 mm x 3.5 mm. The wear loss reading had a standard deviation of 0.4–0.6 mg. The effect of NAP and MWCNTs on wear properties of composite material was determined using the design of experiments (DoE) based L_16_ orthogonal array [[29], [30], [31]]. The experimental factors and their respective levels used for experimentation are shown in Table 5.

**Table 4:** Pin on Disc machine specification.

Parameter	Units
Pin size	8–12 mm
Disc size	50–140 mm
Disc rotation speed	1–2950
Wear track diameter	50–100 mm
Load	5–200N (any steps possible)
Sliding speed range	0-10/s
Power	230V, 50 Hz S phase

**Table 5:** Factors and levels for L_16_ Orthogonal array.

Factors	Level 1	Level 2	Level 3	Level 4
Composition (gms)	24	48	72	96
Load (Kgs)	1	2	3	4
Speed (m/s)	2	3	4	5
Sliding Distance (m)	500	1000	1500	2000

### 2.6 Dynamic mechanical analysis (DMA)

The composite material properties are generally evaluated using parameters, such as frequency, temperature, time, stress, etc. A dynamic test machine is commonly used for this purpose. Dynamic mechanical analysis (DMA) is an approach that deforms the material cyclically permitting the material to react to stress, temperature, frequency, and other variables of the study. The term DMA is also generally referred to as an analyzer for conducting such tests. The DMA equipment with key specifications shown in Table 6, is firmly attached with the composite sample. To obtain the appropriate temperature settings, the DMA equipment is equipped with a temperature-controlled chamber. The DMA instrument monitors the mechanical responses of the composite when dynamic force is applied. The resulting responses represent the sample's strain or displacement as well as the related stress or force. The DMA test data is then evaluated and interpreted to determine critical attributes, such as storage modulus (elastic component), loss modulus (viscous component), and damping properties (tan delta). The data is analyzed and studied to make inferences regarding the viscoelastic behavior and performance of the composite under various settings.

**Table 6:** DMA machine specification.

SI. No.	Parameters	Values & units
1	Maximum force	17 N
2	Force resolution	0.0001
3	Modulus range	10^3^ to 4 × 10^12^ Pa
4	Tan (δ) resolution	0.00001
5	Frequency range	0.01–200 Hz
6	Temperature range	−175 °C–650 °C
7	Heating rate	0.2–25 °C/min

## 3 Results and discussion

The performance of materials in practical applications is typically determined by their physical and mechanical properties. The properties of NAP and MWCNTs are already listed in Table 2. Various tests have been conducted to determine the changes in filler and fiber content, and their effect on the physical and mechanical properties of the manufactured composites.

### 3.1 Variation of tensile, impact and hardness properties for elevated temperatures

**Fig. 2:**
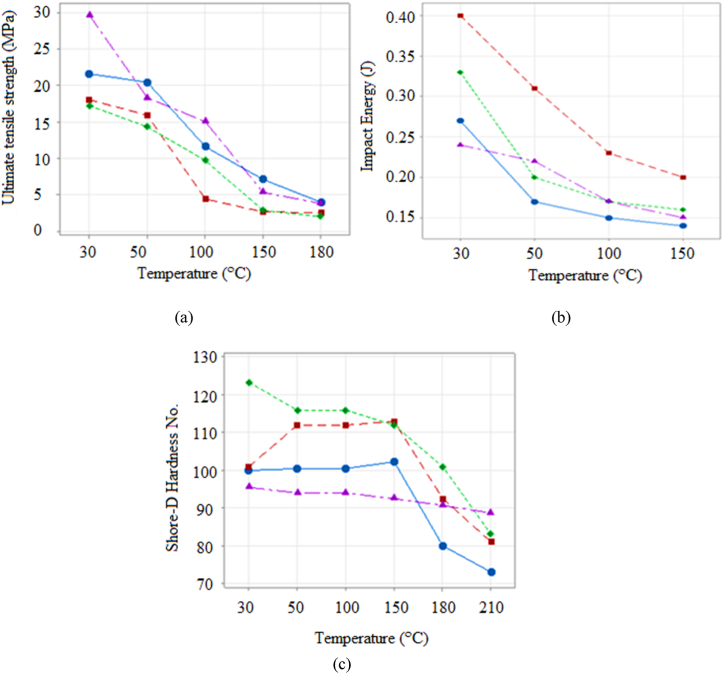
Variation of (a) ultimate tensile strength (UTS), (b) Impact energy, (c) Shore-D hardness of EBH Composites with elevated temperature for 2 % MWCNTs, 5 % NAP, 20 % GF; 2 % MWCNTs, 10 % NAP, 15 % GF; 2 % MWCNTs, 15 % NAP, 10 % GF, 2 % MWCNTs, 20 % NAP, 5 % GF.

From Fig. 2(a), it can be seen that for the composite made of 2 % of MWCNTs, 5 % of NAP, and 20 % GF at room temperature, the tensile strength was found to be 29.64 MPa when compared to its tensile strength at the maximum temperature of 180^o^C which was 3.82 MPa. Also, when the percentage of NAP was constantly increased by 5 % and decreased the percentage of GF by 5 %, it was found that the tensile strength decreased gradually. As a result, as the NAP content does not offer equivalent reinforcing, when the GF content reduces, the total macroscopic tensile strength of the composite is expected to decrease [32,33]. However, because of the additional reinforcing, low amounts of NAP inclusion can sometimes improve tensile strength in some circumstances [33]. This improvement, nevertheless, is only possible if the nanofillers have high tensile strength and excellent interfacial interaction with the matrix. The impact test (Fig. 2(b)) conducted on the manufactured composite for the same composition, i.e., 2 % of MWCNTs, 5 % of NAP, and 20 % GF at room temperature, was found to be 0.24J, and as it reached peak temperature, the impact strength decreased drastically. However, as the percentage of NAP increased and GF decreased by 5 %, the impact strength increased as well. Increasing the NAP concentration can result in enhanced dispersion and interfacial interaction with the matrix. This improves the toughness and energy dissipation, hence increasing the impact strength [33,34]. Except for high temperatures, no significant changes in impact strengths are detected. It is also worth noting that a high NAP concentration decreases the ductility of the composite, making it brittle [24]. Brittle materials have lower impact strength because they are more likely to fracture when subjected to abrupt impact loads [24]. This finding, however, was not observed in the investigated composites. This is mainly due to the interactions between components that enhance overall properties, compensating for increased brittleness, and the NAP that induce structural changes preventing crack propagation, improving impact resistance. The Shore-D hardness test (Fig. 2(c)) conducted for the same composition at room temperature was 95.4 and 88.7 at 180^o^ C. For an increase in the NAP content, the hardness number increased significantly. It is important to note that proper processing and good dispersion of the nanofillers are crucial to achieve consistent hardness [23,24]. Also, the combination of glass fibers and nanofillers tend to have synergistic effects on hardness. This is due to the presence of both types of reinforcements which often lead to a more complex microstructure, resulting in increased hardness [35].

The mechanical properties of the composite are obviously impacted by temperature, since they have all been found to diminish with rising temperatures. It is essential to note that the composites degrade thermally at elevated temperatures. As a result, the tested composites' strength, stiffness, and hardness have all decreased. Furthermore, at high temperatures, the interfacial bonding around the nanoscale reinforcements and the matrix weakens [33,34]. Nanoscale reinforcements might phase split from the matrix under such conditions. Because of the deteriorating bonding, load transmission throughout the phases is limited, leading to poor mechanical performance [33].

### 3.2 Effect of NAP and MWCNTs on wear properties of composite

**Fig. 3:**
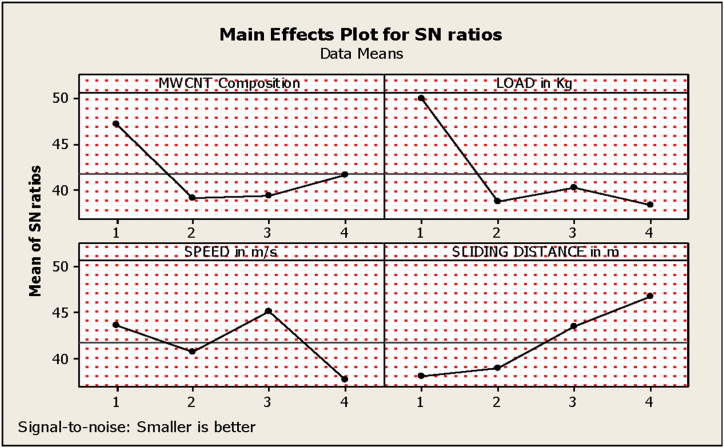
Main effects plot for the wear test.

The experiments for determining wear loss of the manufactured composites were conducted as the L_16_ orthogonal array according to the factors and levels mentioned earlier in Table 5. The experimental results obtained were then incorporated into the statistical Minitab 21 software, and the main effect plots for signal-to-noise ratio (S/N) were developed. The condition for S/N was maintained at ‘Smaller-the-better’. According to Fig. 3, the factor ‘speed’ was seen to have the least impact on wear loss of the composite material. The optimal level for a factor is the level that yields the lowest values within the investigational region. Analyzing the information provided in Fig. 3, it is observed that when the NAP content is 5 %, the material exhibited the highest wear loss, while compositions with higher percentages of NAP experienced less wear loss. This was mainly to the NAP's small size, enabling them to scatter equally inside the composite matrix, resulting in enhanced and improved load transmission during wear. This reinforcement reduces wear mechanisms, such as abrasion, adhesion, and fatigue, resulting in increased durability. The composite demonstrates maximum wear loss when the MWCNTs content is less, but it holds its behavior at higher concentrations and exhibits the least wear loss. MWCNTs generally enhance load transfer and stress distribution throughout the composite matrix during wear [27]. The load transfer mechanism is less efficient at lower concentrations, resulting in greater wear. The material exhibits excellent wear resistance with developed percentages of GFs and at higher speeds. Therefore, the material possesses exceptional wear resistance at elevated speeds. This is because glass fibers (GFs) act as load-bearing elements, distributing loads and inhibiting the progression of fractures during wear. Furthermore, the relative sliding motion between the composite and its opposite surface is more rapid at greater speeds [27]. This minimizes the interaction time between the mating surfaces during wear, giving wear mechanism a shorter time to operate. Wear loss increases as the sliding distance increases, indicating that the material exhibits the least resistance at longer sliding distances [28]. This is due to the debris produced by material removal from both sides amid sliding. More wear debris is formed and deposited at the interface as the sliding distance increases, acting as an abrasive, accelerating the wear process, and resulting in higher wear loss.

### 3.3 Effect of NAP and MWCNTs on dynamic mechanical (DMA) analysis properties of Composite

**Fig. 4:**
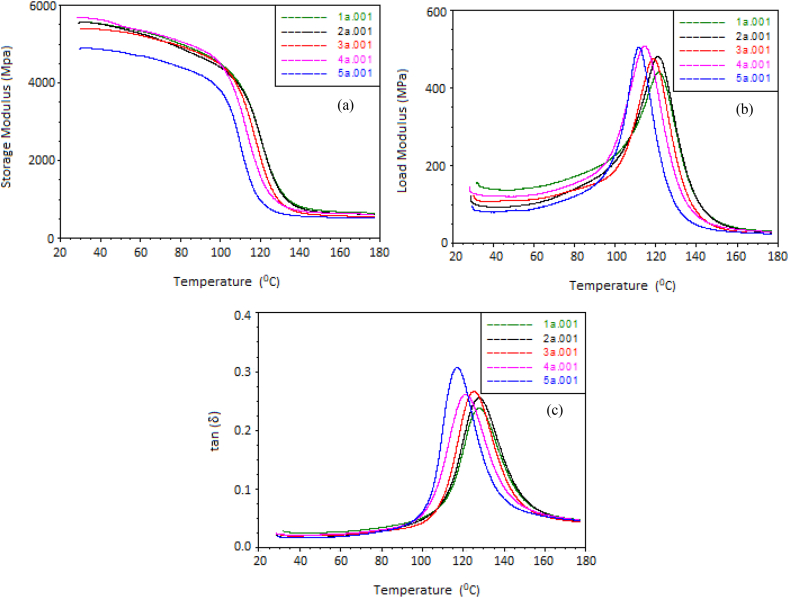
Effect of (a) storage modulus, (b) load modulus, and (c) tan (δ) vs Temperature for **1a**-composition of plate 1 with 2 % MWCNTs, 5 % NAP, 20 % GF; **2a**-composition of plate 2 with 2 % MWCNTs, 10 % NAP, 15 % GF; **3a**-composition of plate 3 with 2 % MWCNTs, 15 % NAP, 10 % GF; **4a**-composition of plate 4 with 2 % MWCNTs, 20 % NAP, 5 % GF; **5a**-composition of plate 5 with 2 % MWCNTs, 0 % NAP, 10 % GF.

A DMA technique was employed to conduct three-point bending measurements, quasi-static micro indentations, and dynamic micro indentations. For the micro indentations, specialized DMA indentor holders were utilized, with a tungsten needle and diamond indentor being employed. Fig. 4 presents results of DMA analysis conducted with different parameters for composite materials of varying compositions. The dynamic mechanical properties of the composites, reinforced with various materials, were successfully examined at different frequencies, leading to the following findings. Fig. 4(a) illustrated the changes in the storage modulus of the composites as a function of temperature, while maintaining constant frequencies, for different compositions. The storage modulus of a composite generally increases when the NAP content is increased as they contribute to the reinforcement of the material, leading to an increase in stiffness and mechanical properties. The storage modulus also shows a reverse effect at a temperatures beyond 100^o^C, which might be due to weakness developed in interfacial bonding at

Elevated temperature [34,35]. Like the storage modulus results, the compositions also exhibited noticeable variations in the load moduli of the composites, as shown in Fig. 4(b). It was observed that the peaks in the loss modulus curve increased with higher percentages of NAP. Particularly, the composite with the composition of Specimen 4, consisting of 2 % MWCNTs, 20 % NAP, and 5 % GF, displayed the highest peak in the load modulus curve. The increase in peak values of the load modulus curve is attributed to the improved molecular mobility resulting from increased frequencies [34]. Fig. 4(c) displays the variations in damping properties of the composite as a function of temperature for different compositions. The highest peak in damping was observed in the specimen having MWCNTs and GFs without NAP concentration. This is due to the synergistic effects of both reinforcements, leading to enhanced damping capabilities [36]. Also, GFs provide additional damping characteristics to the composite due to their viscoelastic nature, and MWCNTs distribute mechanical loads, leading to improved energy dissipation during vibrations.

### 3.4 Surface morphologies of the composites

**Fig. 5:**
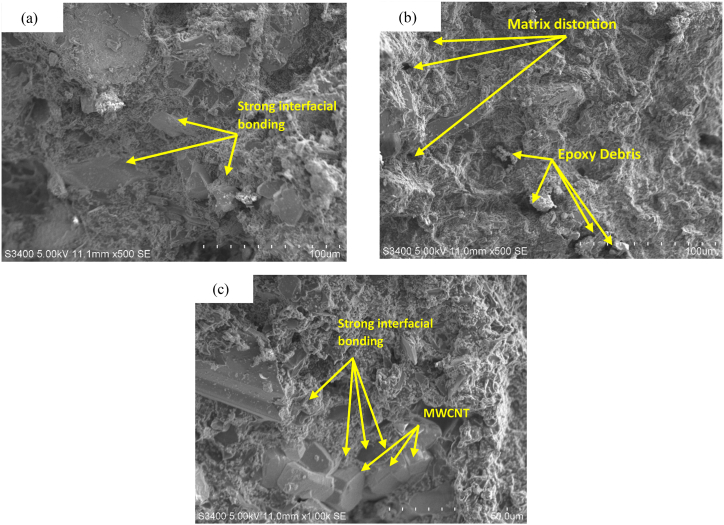
SEM images of the fracture surface of tensile specimens for (a) 2 % MWCNTs, 10 % NAP, 15 % GF, (b) 2 % MWCNTs, 15 % NAP, 10 % GF, and (c) 2 % MWCNTs, 20 % NAP, 5 % GF.

The interfacial bonding between the fiber and matrix may be significantly affected by the state of dispersion of MWCNTs and NAP on the fiber surface. The surface morphologies of the fracture surface of the tensile tested samples have been investigated by SEM to confirm the effect of MWCNT and NAP concentrations on the interfacial characteristics of composites, as illustrated in Fig. 5. It has already been determined that the tensile strength of the fabricated composites was enhanced by adding the NAP. According to Fig. 5(a) and (b), a substantial number of epoxy matrices appear to stay adherent to the surface of multiscale composites composed of MWCNTs, NAP, and GF, and no debonding of the matrix from the fibers, nanotubes, and fillers was detected. More epoxy ruptures and matrix distortion were detected on the fractured surface of 2 % MWCNTs, 10 % NAP, and 15 % GF (Fig. 5(b)) as opposed to 2 % MWCNTs, 10 % NAP, and 15 % GF (Fig. 5(a)). This is because of the existence of increased NAP% in the epoxy matrix alongside MWCNT, which improves load transmission between the GF and epoxy matrix, offering a larger surface area to the MWCNT [35]. As shown in Fig. 5(c), the MWCNTs and a large proportion of NAP are tightly adherent to the matrix surface, showing strong interfacial adhesion and complete bonding of the matrix and fiber. These results show that MWCNTs and NAP surrounding GFs could lock with epoxy matrices to promote multiscale composite interfacial adhesion. The MWCNTs and NAP at the fiber-matrix interface operate as crack arresters, preventing the spread of microcracks. Also, excellent MWCNT and nanoparticle dispersion and excellent interfacial adhesion might promote energy dissipation throughout matrix deformation and postpone shear failure.

**Fig. 6:**
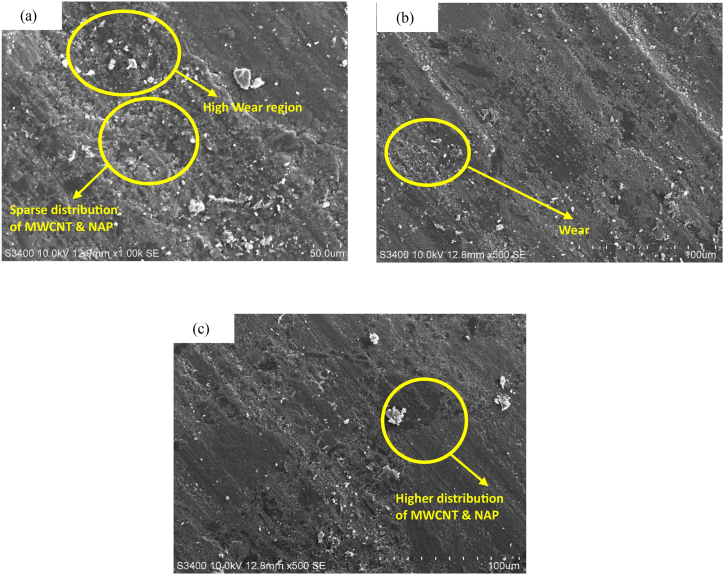
SEM images of the worn surfaces for (a) 2 % MWCNTs, 10 % NAP, 15 % GF, (b) 2 % MWCNTs, 15 % NAP, 10 % GF, and (c) 2 % MWCNTs, 20 % NAP, 5 % GF.

The wear loss of the produced composites was evaluated at 10N normal load and 3 m/s sliding velocity, as shown in Fig. 6. The wear loss was reduced by the incorporation of MWCNTs and NAP (Fig. 6(a, b, and c)). This decrease is caused by the fact that when the surface asperities on the composite are distorted, the true area of engagement increases, resulting in a decrease in pressure. The maximal network produced by MWCNTs, and NAP is responsible for this result. In fact, MWCNTs and NAP provide a high aspect ratio, strong reinforcement, and superior load transferability in composites via a stronger interface encourages high property [27,28]. As a result, deforming the surface of such composites requires a lot of energy. Furthermore, the composite surface comprises MWCNTs, which are thermally more stable than the clean composite, and their high aspect ratio increases thermal conductivity [28]. The presence of MWCNT acts as a reinforcing phase wherein load transfer across the matrix and the reinforcing elements improves resulting in the overall enhancement of nanocomposites wear resistance. It also improves the interfacial bonding between the matrix, GFs, and nanoparticles resulting in the reduction of localized wear. The localized wear tends to highly influence the nature and size of the wear debris, as the wear debris is smaller, contributing to the wear process [37].

## 4 Conclusions

The present study explored the mechanical properties of a composite material composed of multi-walled carbon nanotubes (MWCNTs), nano aluminum powder (NAP), and glass fibers (GF). The impact strength, ultimate tensile strength (UTS), hardness, along with the dynamic mechanical analysis (DMA) of the composites were evaluated at elevated temperatures. The analysis of results led to the following conclusions.

• The composite with a composition of 2 % MWCNTs, 15 % NAP, and 10 % GF exhibited the highest impact strength, as indicated by the maximum impact energy.
• The ultimate tensile strength (UTS) at room temperature was found to be for the composition having 2 % MWCNTs, 5 % NAP, 20 % GF, due to the presence of GFs and NAP in good proportions.
• The hardness of the composite was highest for the composition having 2 % MWCNTs, 20 % NAP, 5 % GF, due to the high presence of NAP at room temperature.
• A higher percentage concentration of NAP in the composite decreased the wear loss significantly due to its small size and uniform dispersion throughout the matrix, leading to improved load distribution during wear.
• The surface morphologies showed that the MWCNTs along with a higher percentage of NAP, firmly adhere to the surface of the matrix, indicating strong interfacial adhesion and the complete bonding between the fiber and the matrix.
• Overall, the findings from the tests suggest that increasing the amount of GF and NAP enhances the mechanical properties of the composite. Moreover, the inclusion of other fillers, such as calcium carbonate, kaolin (hydrous aluminum silicate), aluminum trihydrate, calcium sulfate, among others, allows for the modification of the composite based on its intended application.

## Disclosure of interest

The authors declare that they have no known competing financial interests or personal relationships that could have appeared to influence the work reported in this paper.

## Declaration of funding

No external funding was received for the research conducted in this study.

## Data and code availability

Data included in article/supplementary material is referenced in the article.

## CRediT authorship contribution statement

**Mohammed Nadeem M:** Writing – original draft, Methodology, Data curation, Conceptualization. **Jaimon Dennis Quadros:** Writing – review & editing, Formal analysis, Data curation, Conceptualization. **Yakub Iqbal Mogul:** Writing – review & editing, Methodology, Investigation, Conceptualization. **Ma Mohin:** Writing – original draft, Methodology, Formal analysis. **Abdul Aabid:** Writing – review & editing, Formal analysis, Data curation. **Muneer Baig:** Writing – review & editing, Funding acquisition. **Omar Shabbir Ahmed:** Funding acquisition, Formal analysis.

## Declaration of competing interest

The authors declare the following financial interests/personal relationships which may be considered as potential competing interests.
